# Sex‐dependent jugular vein optical attenuation and distension during head‐down tilt and lower body negative pressure

**DOI:** 10.14814/phy2.15179

**Published:** 2022-02-12

**Authors:** Courtney A. Patterson, Robert Amelard, Essi Saarikoski, Hannah Heigold, Richard L. Hughson, Andrew D. Robertson

**Affiliations:** ^1^ Schlegel‐University of Waterloo Research Institute for Aging Waterloo Ontario Canada; ^2^ Department of Kinesiology University of Waterloo Waterloo Ontario Canada; ^3^ KITE‐Toronto Rehabilitation Institute University Health Network Toronto Ontario Canada; ^4^ Department of Systems Design Engineering University of Waterloo Waterloo Ontario Canada

**Keywords:** fluid shift, internal jugular vein, non‐contact optical imaging, venous congestion

## Abstract

Non‐contact coded hemodynamic imaging (CHI) is a novel wide‐field near‐infrared spectroscopy system which monitors blood volume by quantifying attenuation of light passing through the underlying vessels. This study tested the hypothesis that CHI‐based jugular venous attenuation (JVA) would be larger in men, and change in JVA would be greater in men compared to women during two fluid shift challenges. The association of JVA with ultrasound‐based cross‐sectional area (CSA) was also tested. Ten men and 10 women completed three levels of head‐down tilt (HDT) and four levels of lower body negative pressure (LBNP). Both JVA and CSA were increased by HDT and reduced by LBNP (all *p* < 0.001). Main effects of sex indicated that JVA was higher in men than women during both HDT (*p* = 0.003) and LBNP (*p* = 0.011). Interaction effects of sex and condition were observed for JVA during HDT (*p* = 0.005) and LBNP (*p* < 0.001). We observed moderate repeated‐measures correlations (*r*_rm_) between JVA and CSA in women during HDT (*r*_rm_ = 0.57, *p* = 0.011) and in both men (*r_r_*_m_ = 0.74, *p* < 0.001) and women (*r*_rm_ = 0.66, *p* < 0.001) during LBNP. While median within‐person correlation coefficients indicated an even stronger association between JVA and CSA, this association became unreliable for small changes in CSA. As hypothesized, JVA was greater and changed more in men compared to women during both HDT and LBNP. CHI provides a non‐contact method of tracking large changes in internal jugular vein blood volume that occur with acute fluid shifts, but data should be interpreted in a sex‐dependent manner.

## Graphical abstract

Optical attenuation of near‐infrared light over the internal jugular vein is associated with cross‐sectional area during fluid shifts instigated by head‐down tilt (pictured) and lower body negative pressure. Men had greater optical attenuation across all conditions while women had larger cross‐sectional areas than men. Greater changes in optical attenuation in men than women across all conditions need to be accounted for in research and potential clinical applications of this technique.

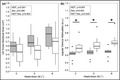

## 1 INTRODUCTION

Internal jugular vein (IJV) volume is an important indicator of central blood volume and venous congestion in physiological manipulations, spaceflight, and pathological conditions such as heart failure (Arbeille et al., 2015; Pellicori et al., 2021; Wang et al., 2020). Regular monitoring of IJV volume may help guide treatment of hypovolemic and hypervolemic conditions (Antonelli et al., 2007; Pellicori, Kaur, et al., 2015). The most common surrogate for IJV volume is vessel cross‐sectional area (CSA) measured with 2D ultrasound, though 3D ultrasound techniques have also been developed (Arbeille et al., 2017; Groves et al., 2020). Importantly, however, while venous ultrasound is non‐invasive, its reproducibility is sensitive to anatomical location (Wang et al., 2020) and hold down pressure, thus requiring a trained operator and limiting the clinical utility to acute‐care settings. Recent advances in optical imaging have enabled non‐contact jugular venous assessment to help alleviate these barriers.

Coded hemodynamic imaging (CHI) is a wide‐field near‐infrared camera system that quantifies blood volume in shallow‐to‐medium depth vessels (Amelard et al., 2017, 2021). CHI measures optical attenuation derived from the amount of light absorbed by hemoglobin and other underlying chromophores (e.g., myoglobin, melanin; Jacques, 2013). As hemoglobin is the main time‐varying chromophore associated with hemodynamic profiles, vascular metrics, such as the IJV pulse waveform, can be derived from the time‐varying signal proportional to jugular vein optical attenuation (JVA; Amelard et al., 2017, 2021). Vascular responsiveness to acute changes in blood volume distribution can be assessed by altering the orthostatic gradient. Head‐down tilt (HDT), for example, induces a cephalad fluid shift resulting in increased IJV blood volume (Petersen et al., 2016). Conversely, central hypovolemia can be simulated using lower body negative pressure (LBNP; Cooke et al., 2004) to translocate blood from the central circulation to the lower body, resulting in reduced central venous volume and pressure (Edgell et al., 2012). Using CHI, JVA was previously shown to be strongly associated with central venous pressure during both HDT and LBNP conditions (Amelard et al., 2021). However, non‐contact optical imaging has not yet been compared against traditional ultrasound measures of IJV blood volume.

Coded hemodynamic imaging is currently limited to tracking relative changes in JVA within an individual, while absolute absorption levels will vary due in part to individual differences in hemoglobin, as well as the size and depth of other underlying tissues—attributes with known sex‐specific distributions (Murphy, 2014; Nagai et al., 2020). Sex‐based differences in CSA are equivocal (Jeon et al., 2020; Magnano et al., 2016), although there is some indication that men have larger right IJV than women (Magnano et al., 2016), while IJV distensibility appears similar between men and women (Mortensen et al., 1990). In contrast, hemoglobin concentrations (Murphy, 2014) and sternocleidomastoid muscle volume (Nagai et al., 2020) are known to be greater in men. These differences, as well as possible sex differences in CSA, may confound the utility of CHI to monitor IJV blood volume between sexes. This study aimed to understand sex‐specific responses of JVA by relating the observed changes to ultrasound imaging of the contralateral IJV during acute fluid shifts. Specifically, we assessed the association between JVA and CSA during multiple levels of HDT and LBNP in healthy young men and women. Due to the established sex differences in hemoglobin concentration and sternocleidomastoid thickness, we hypothesized that men would have a greater JVA than women, and that the change in JVA in response to HDT and LBNP would be greater in men than women.

## 2 MATERIALS AND METHODS

### 2.1 Participants and protocol

Participant characteristics and experimental protocol were described in detail previously (Amelard et al., 2021). Briefly, 20 young healthy adults (10 women) with no history of vascular, inflammatory, or thrombotic disease were recruited. The study was approved by a University of Waterloo Research Ethics Board (ORE #40394) and all protocols conformed to ethical principles set forth in the Declaration of Helsinki, with exception of registration in a database. All participants provided written informed consent prior to testing. Participants were asked to abstain from caffeine, nicotine, alcohol, and heavy exercise for 24 h prior to the study. Furthermore, participants fasted for 2 h and were asked to consume ~500–1000 ml of water 2 h prior to participation. Testing occurred between 09h00 and 17h00. Participants underwent three levels of HDT (0, −3, and −6°) and four levels of LBNP (0, −20, −30, and −40 mmHg). The order of the HDT and LBNP was randomized per participant, with at least 5 min between conditions, and the levels within each condition were conducted in sequence with a duration of ~5 min per level. LBNP was discontinued early if systolic blood pressure dropped below 70 mmHg, which occurred in one participant at LBNP −40 mmHg.

### 2.2 Anthropometrics

Height and weight were measured directly. Body mass index and body surface area (Du & Bois, 1916) were calculated. The left IJV depth and sternocleidomastoid muscle cross‐sectional thickness were measured during supine rest from ultrasound imaging.

### 2.3 Ultrasonography

The left IJV was imaged by an experienced sonographer (Andrew Donald Robertson) at each level of each condition after at least 1 min of stabilization. The IJV was measured inferior to the midpoint along a direct line from the sternal notch to the mastoid process. A 5‐s cine loop was acquired of the vessel cross‐section using a 9–3 MHz linear array probe (iE33 xMatrix; Koninklijke Philips N.V.). Offline, individual frames were extracted from the exported video file (~23 fps, VLC Media Player; https://www.videolan.org/vlc/index.html). The frame immediately following each ECG R‐spike was used for analysis, approximately corresponding with the c‐wave of the jugular venous pulse. The CSA was quantified semi‐automatically using the ellipse tool in ImageJ (https://imagej.nih.gov/ij/) and adjusted manually. The maximum CSA, across ~5 cardiac cycles, at each level of each condition was used for analysis to minimize variability due to respiration.

### 2.4 Coded hemodynamic imaging

The CHI system, which uses a 4.2‐megapixel camera, was positioned to provide orthogonal illumination of the right lateral side of the neck and was moved with the participant between conditions in order to minimize deviations in field of view and angle of illumination. The system used a 940‐nm near‐infrared light‐emitting diode (LZ1‐10R702, LED Engin; OSRAM Sylvania Inc.) and cross‐polarization optics for deep tissue penetration (Amelard et al., 2021). A 30‐s video (60 fps) was acquired for each level of each condition immediately following the ultrasound acquisition. A finger photoplethysmography signal was acquired simultaneously for use in CHI processing.

A 4 × 4 mean pixel down‐sampling of the reflected light was performed to increase the signal‐to‐noise ratio of the spatiotemporal signals. The resulting diffuse reflectance map was denoised with a hemodynamic Kalman filter process and calibrated using a flexible reflectance target to calculate optical attenuation, as described previously (Amelard et al., 2021). To localize the IJV pulse, a spatial map correlating the attenuation signal at each down‐sampled pixel against the time‐synchronized photoplethysmography waveform was examined for regions of inverse correlation to identify venous pulsatility (Amelard et al., 2017). The IJV was targeted as the largest region of contiguous pixels inferior from the midline of the neck showing inverse correlation. JVA was extracted as the mean attenuation across the contiguous IJV region of interest found approximately in the same ~1 cm segment of the vessel in each participant. The peak JVA across each 30‐s acquisition was used for analysis to minimize variability due to respiration. One female participant's data were removed due to an insufficient area for imaging, and one male participant's LBNP data were removed due to acquisition issues.

### 2.5 Statistical analysis

Statistical analysis was performed using R (R Core Team, 2015) with figures generated using ggplot (tidyverse library; Wickham et al., 2019). Descriptive statistics are reported as mean ± *SD*. Sex differences in demographic data were assessed using Student's *t*‐tests. Pearson correlations were used to determine the association between JVA and sternocleidomastoid thickness, as well as JVA and IJV depth. We used separate linear mixed models to assess the effects of sex and condition (HDT or LBNP), as well as their interaction, on CSA and JVA. Sex and condition were entered as fixed effects, and participant was entered as a random effect (lme4 library; Bates et al., 2015). To assess whether the influence of sex was directly a result of sex‐based differences in baseline CSA, these models were also tested with baseline CSA included as a covariate. Post hoc analysis used pairwise comparison of the estimated marginal means with Tukey adjustment for multiple comparisons (emmeans library; Lenth, 2021). The association between JVA and CSA was assessed for HDT and LBNP, as well as for men and women, separately. A repeated‐measures correlation (*r*_rm_) analysis, which assesses the fit of a common slope across all participants, was completed to examine for a collective underlying association between the two variables within each group (rmcorr library; Bakdash & Marusich, 2021). Subsequently, individual regression models between JVA and CSA were completed for each participant to account for between‐participant vascular and tissue differences. Pearson correlation coefficients are reported as the median (*r*_median_) and interquartile range (IQR) for each group. The individual regression slopes separated by condition and sex were compared using a Wilcoxon rank sum test and are reported as median and IQR.

## 3 RESULTS

Participant demographics separated by sex are presented in Table 1. No differences between men and women were observed for age (*p* = 0.521) or body mass index (*p* = 0.213), but men had significantly greater body surface area (*p* < 0.001), sternocleidomastoid thickness (*p* = 0.003), and IJV depth (*p* < 0.001) compared to women. At baseline (HDT 0°), men and women did not have a significant difference in CSA (*p* = 0.117), but men had a significantly greater JVA compared to women (*p* = 0.015). Across men and women, JVA was weakly associated with sternocleidomastoid thickness (*r* = 0.43, *p* < 0.001) and depth to IJV (*r* = 0.36, *p* < 0.001).

**TABLE 1:** Participant demographics for women and men

Characteristic	Women (*n* = 9)	Men (*n* = 10)	*p*‐value
Age (years)	24 ± 4	25 ± 5	0.521
Body surface area (m^2^)	1.61 ± 0.16	1.90 ± 0.15	<0.001
Body mass index (kg/m^2^)	22.2 ± 2.1	23.7 ± 3.1	0.213
CSA (cm^2^)	0.60 ± 0.18	0.44 ± 0.25	0.117
JVA (a.u.)	0.51 ± 0.07	0.61 ± 0.09	0.015
Sternocleidomastoid thickness (cm)	0.81 ± 0.14	1.13 ± 0.23	0.003
Depth to IJV (cm)	1.17 ± 0.12	1.51 ± 0.21	<0.001

Note

Values are mean ± *SD*. CSA and JVA are measured while lying supine during the head‐down tilt protocol (0°).

Abbreviations: CSA, cross sectional area; IJV, internal jugular vein; JVA, jugular venous attenuation.

### 3.1 CSA and JVA response to HDT

**FIGURE 1:**
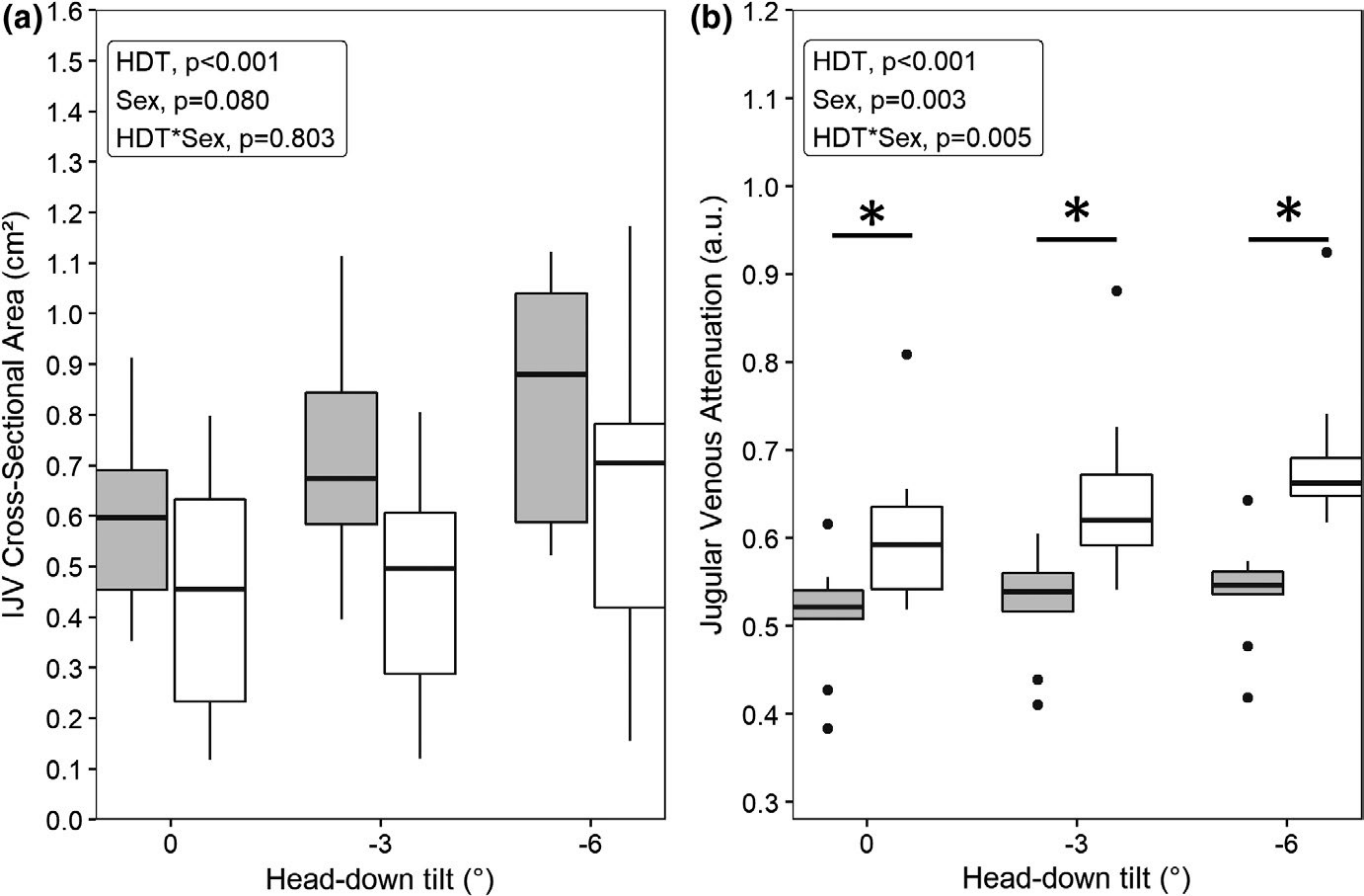
Change in internal jugular vein (IJV) cross‐sectional area (a) and optical attenuation (b) across three levels of head‐down tilt (HDT) separated by women (grey, *n* = 9) and men (white, *n* = 10). Statistics were performed using linear mixed‐effects models (variable ~ HDT level × sex +1|participant). *Significant difference (*p* < 0.05) between women and men. Boxplot description: center lines indicate medians, bottom and top edges indicate first and third quartiles, and whiskers indicate the entire range of data without outliers (dots); outliers were identified as >1.5 times the interquartile range beyond the top or bottom edge of each box

Internal jugular vein CSA was increased by HDT (main effect of HDT, *p* < 0.001). CSA tended to be greater in women (main effect of sex, *p* = 0.080), but the change in CSA across levels of HDT was not affected by sex (interaction effect, *p* = 0.804; Figure 1a). Subsequently, controlling for differences in baseline CSA did not alter the results. JVA was also increased by HDT (main effect of HDT, *p* < 0.001). Moreover, JVA was greater in men than women (main effect of sex, *p* = 0.003) and the increase in JVA with HDT was greater in men than women (interaction effect, *p* = 0.005; Figure 1b). Men had a significantly greater JVA at 0° HDT (*p* = 0.017), −3° HDT (*p* = 0.003), and −6° HDT (*p* = 0.001; Figure 1b). When controlling for baseline CSA in the JVA model, the main effects of HDT (*p* < 0.001) and sex (*p* = 0.006), as well as their interaction (*p* = 0.005) remained significant.

### 3.2 CSA and JVA response to LBNP

**FIGURE 2:**
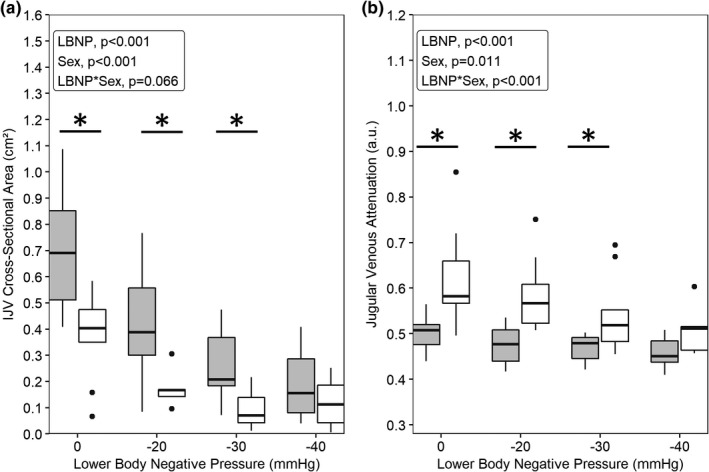
Change in internal jugular vein (IJV) cross‐sectional area (a) and jugular venous attenuation (b) across four levels of lower‐body negative pressure (LBNP) separated by women (grey, *n* = 9) and men (white, *n* = 9). Statistics were performed using linear mixed‐effects models (variable ~ LBNP level × sex +1|participant). *Significant difference (*p* < 0.05) between men and women. Boxplot description: center lines indicate medians, bottom and top edges indicate first and third quartiles, and whiskers indicate the entire range of data without outliers (dots); outliers were identified as >1.5 times the interquartile range beyond the top or bottom edge of each box

Cross‐sectional area was reduced by LBNP (main effect of LBNP, *p* < 0.001). Women had a larger CSA during the LBNP protocol (main effect of sex, *p* < 0.001), and the decrease in CSA with LBNP tended to be larger in women than men (interaction effect, *p* = 0.066; Figure 2a). CSA was significantly larger in women at 0 mmHg (*p* = 0.001), −20 mmHg (*p* = 0.005), and −30 mmHg (*p* = 0.032), but not at −40 mmHg (*p* = 0.094; Figure 2a). When controlling for baseline CSA, the sex‐by‐LBNP interaction effect remained a significant trend (*p* = 0.064) and the main effect of sex was attenuated but still significant (*p* = 0.042). In contrast to CSA, men had a larger JVA during LBNP (main effect of sex, *p* = 0.011). JVA decreased during LBNP (main effect of level, *p* < 0.001) and the reduction in JVA during LBNP was greater in men than women (interaction effect, *p* < 0.001; Figure 2b). JVA was significantly greater in men at 0 mmHg (*p* = 0.001), −20 mmHg (*p* = 0.005), and −30 mmHg (*p* = 0.032), but not at −40 mmHg (*p* = 0.094; Figure 2b). When controlling for baseline CSA in the JVA model, the main effects of LBNP (*p* < 0.001) and sex (*p* = 0.034), as well as their interaction (*p* < 0.001) remained significant.

### 3.3 Association between JVA and CSA

**FIGURE 3:**
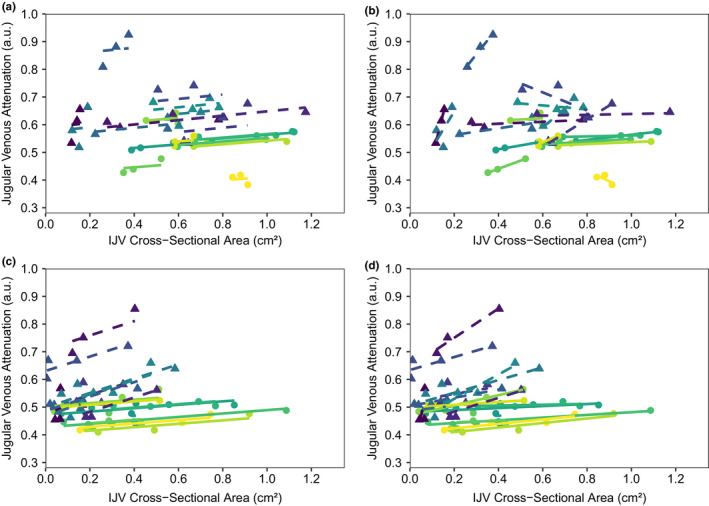
Association between jugular venous attenuation and internal jugular vein (IJV) cross‐sectional area during head‐down tilt (HDT; a,b) and lower body negative pressure (LNBP; c,d). (a) Repeated‐measures correlations with common slope during HDT for men [*r*_rm_(95% CI) =0.26 (−0.03, 0.55), *p* = 0.263] and women [*r*_rm_(95% CI) =0.57 (0.31, 0.78), *p* = 0.011], separately. (b) Individual regressions per participant during HDT for men [*r*_median_ (IQR) =0.70 (0.51, 0.99)] and women [*r*_median_ (IQR) =0.91 (0.75, 1.00)]. (c) Repeated‐measures correlations with common slope during LBNP for men [*r*_rm_(95% CI) =0.74 (54, 0.88), *p* < 0.001] and women [*r*_rm_(95% CI) =0.66 (0.43, 0.81), *p* < 0.001], separately. (d) Individual regressions per participant during LBNP for men [*r*_median_ (IQR) =0.83 (0.58, 0.99)] and women[*r*_median_ (IQR) =0.85 (0.39, 0.98)]. Data points are from three HDT angles and four LBNP levels, and each participant is represented by a different colour. In each plot, men (*n* = 10) are shown using triangles and dashed regression lines, and woman (*n* = 9) are shown using circles and solid regression lines. IQR, interquartile range

Across all levels of HDT, repeated‐measures correlation revealed a moderate association between JVA and CSA for women [*r*_rm_(95% CI) = 0.57 (0.31, 0.78), *p* = 0.011], but no association for men [*r*_rm_(95% CI) = 0.26 (−0.03, 0.55), *p* = 0.263] (Figure 3a). The common slope was 0.08 a.u./cm^2^ for men and 0.06 a.u./cm^2^ for women. Using individual regression models for each participant, a stronger association between JVA and CSA was observed for both men [*r*_median_ (IQR) = 0.70 (0.51, 0.99)] and women [*r*_median_ (IQR) = 0.91 (0.75, 1.00)] (Figure 3b). No difference in individual regression slope between men and women was observed [median (IQR) = 0.31 (0.02, 0.92) vs. 0.04 (0.03, 0.14) a.u./cm^2^; *p* = 0.356), although significant variability was observed.

Across all levels of LBNP, repeated‐measures correlation revealed a strong association between JVA and CSA for men [r_rm_(95% CI) = 0.74 (54, 0.88), *p* < 0.001] and a moderate association for women [*r*_rm_(95% CI) = 0.66 (0.43, 0.81), *p* < 0.001] (Figure 3c). The common slope was 0.26 a.u./cm^2^ for men and 0.06 a.u./cm^2^ for women. Using individual regression models for each participant, a stronger association between JVA and CSA was observed for both men [*r*_median_ (IQR) = 0.83 (0.58, 0.99)] and women [*r*_median_ (IQR) = 0.85 (0.39, 0.98)] (Figure 3d). Men had a significantly larger individual regression slope compared to women [median (IQR) = 0.23 (0.14, 0.44) vs. 0.05 (0.03, 0.08) a.u./cm^2^; *p* < 0.001).

Notably, one man and two women showed an inverse association between the two metrics; all during HDT. All three of these participants had very little change in CSA across the three levels of HDT (<0.15 cm^2^), which may have limited our ability to quantify an association between JVA and CSA. Across both HDT and LBNP data, when the change in CSA was under 0.15 cm^2^, the variability of the individual‐level correlation coefficients between JVA and CSA was very high (IQR = 0.03, 0.84); when the change in CSA was over 0.15 cm^2^, the variability was substantially reduced (IQR = 0.79, 0.99).

## 4 DISCUSSION

The current study showed differences in JVA between men and women both at rest and in response to acute cephalad and caudad fluid shifts. As hypothesized, JVA was greater in men compared to women across all HDT and LBNP conditions (exception at −40 LBNP). Furthermore, the change in JVA across each condition was larger in men. In contrast, absolute CSA and change in CSA tended to be greater in women compared to men. These contrasting responses were further emphasized by differing slopes characterizing the association between JVA and CSA, at least during LBNP. Thus, while moderate‐to‐strong associations between JVA and CSA within conditions demonstrate value for JVA as a non‐contact hemodynamic index, sex‐specific factors appear necessary to fully interpret JVA as a function of blood volume.

Women tended to have a larger CSA compared to men, notably during the LBNP condition. This observation constrasts with previous studies which have reported no significant difference in left CSA between men and women during supine rest (Jeon et al., 2020; Magnano et al., 2016). Larger body mass index has been associated with greater IJV CSA (Jeon et al., 2020; Magnano et al., 2014, 2016); however, men and women in our cohort had similar body mass indexes. Furthermore, while a weak positive correlation between CSA and body surface area has been reported (Mortensen et al., 1990), this explanation does not fit our data. Our observation that women had a greater CSA than men was consistent during each level of LBNP, with the exception of −40 mmHg. No previous study has compared CSA between men and women during acute manipulations of central blood volume; however, the loss of separation at the final stage of LBNP was likely due to a floor effect as the smaller male veins approached collapse prior to those of the women. Between study differences in resting IJV might be the result of the variable geometry along the length of the IJV (Wang et al., 2020) and requires further study. In addition, possible mechanisms for differences observed between men and women in CSA should be further explored, especially considering research has previously suggested distensibility was similar between men and women (Mortensen et al., 1990).

In contrast to the larger CSA measured in women by ultrasound, absolute near‐infrared light absorption quantified by JVA was greater in men than women. While we interpret changes in JVA as a proxy for changes in blood volume due to the absorption characteristics of hemoglobin (Jacques, 2013), differences in baseline JVA could be attributed not only to greater hemoglobin concentration, but also to greater myoglobin concentration and greater vessel depth in men. While we did not measure serum hemoglobin, it is well known that the concentration is ~15% higher in adult men compared to women (Hawkins et al., 1954; Jorgensen et al., 2019). This corresponds well to the 19% larger JVA in men we report at baseline. Future work with JVA should include serum hemoglobin concentration to explore this hypothesis. Myoglobin also absorbs infrared light (Bashkatov et al., 2011; Jacques, 2013) so the sternocleidomastoid muscle could confound the relationship between absorption signals and IJV blood volume in addition to the effects of hemoglobin. We observed the sternocleidomastoid to be significantly thicker in men compared to women, consistent with previous findings (Nagai et al., 2020), and weakly correlated with JVA signal. Importantly, however, while myoglobin within muscle overlying the IJV might contribute to the larger baseline JVA signal in men, it would not account for the greater change in JVA with HDT or LBNP. We also found that the IJV was deeper in men compared to women. In silico modeling demonstrated that vessel depth can impact JVA, which is consistent with the weak correlation observed between IJV depth and JVA at baseline. Overall, these anatomical and physiological sex differences do appear to impact the strength of JVA signal. Sex and optical hemodynamic imaging should be studied in more detail within future studies.

Head‐down tilt redistributes blood from the lower limbs toward the head, resulting in increased CSA of the IJV (Arbeille et al., 2001; Marshall‐Goebel et al., 2018). Increased CSA occurs immediately upon assuming a head down position (Arbeille et al., 2001; Clenaghan et al., 2005; Schreiber et al., 2002) and has been observed to remain enlarged for up to 42 days of continuous HDT (Arbeille et al., 2001). Due to the highly compliant nature of veins, CSA expansion is indicative of increased venous pressure (Chua Chiaco et al., 2013; Dawson et al., 2004; Martin et al., 2016). Elevated IJV pressure in HDT contributes to the hydrostatic gradient that increases intracranial pressure (Petersen et al., 2016). Here, we show that non‐contact optical imaging can track changes in CSA during acute cephalad fluid shifts, thereby quantifying the extent of venous congestion rapidly without the need for contact‐based probes. Although the association between JVA and CSA during venous congestion appears to be stronger in women, this may be related to our findings of larger IJV and thinner overlaying muscle tissue in women.

Lower body negative pressure exerts an opposite effect to that of HDT, passively shifting blood from the central veins to the lower limbs as a model of central hypovolemia and hemorrhagic shock (Cooke et al., 2004). Our observations of reduced CSA with increasing LBNP is consistent with the literature (Petersen et al., 2019; Watkins et al., 2017). The current study provides evidence that CHI can track changes in CSA at LBNPs up to −40 mmHg without the need for skin contact. Thus, this technology may be feasible to quickly assess the effects of central hypovolemia and hemorrhagic shock. Notably, LBNP was terminated early due to low systolic blood pressure in a single participant. Hypotensive signs did not appear until the early stages of −40 mmHg LBNP and JVA could not be measured prior to discontinuation of the protocol. Physiologically, this participant behaved similarly to the group up to −30 mmHg LBNP. Future work to capture this time sensitive period would benefit from CHI acquisition during the early transition to higher orthostatic stresses.

We employed two different statistical models to assess the relationship between JVA and CSA. Repeated‐measures correlation assesses the linear association between two variables that is common within a group by adjusting for between‐person variability using a modified ANCOVA model (Bakdash & Marusich, 2017). In repeated‐measures studies, this technique avoids the pitfalls of Pearson correlation, which assumes independence of errors between paired data points and does not properly account for both within‐ and between‐individual variance (Shan et al., 2020), and precludes the need for data aggregation which may limit statistical power (Bakdash & Marusich, 2017). Within the context of the current work, however, it is important to recognize that repeated‐measures correlation relies on an estimated common slope for all participants upon which the association between the two variables is based, and a divergence from slope homogeneity results in a smaller effect size (Bakdash & Marusich, 2017). Optical hemodynamic imaging outcomes, such as JVA in the current study, are influenced by individual factors such as vessel depth (Martelli et al., 2016), adiposity, and skin tone (Jacques, 2013). The influence of these individual factors leads to between‐person heterogeneity in the association between JVA and CSA, and therefore results in a weaker overall association using the repeated‐measures model. As an alternative, we performed individual participant‐level regressions, which allowed for unique slopes between participants. Among these, the median correlation was greater than the repeated‐measures correlation suggesting that individual factors do influence the JVA measurement on an individual level. This influence is alluded to by the weak associations that were found between JVA and IJV depth, as well as JVA and sternocleidomastoid thickness. Although the median individual correlation coefficients were greater than those from the repeated‐measures analyses, three individuals had negative correlation coefficients resulting from the HDT data. All three of these individuals had small changes in CSA (<0.15 cm^2^) across the HDT condition. In our data, the variability of the individual‐level correlation coefficients between CSA and JVA was very high when the individual change in CSA was under 0.15 cm^2^. This emphasizes the point that JVA may reliably track only large changes in CSA. Prior work has shown group mean increases in CSA of 0.15 to 0.23 cm^2^ in the first 5 min of −5 or −6° HDT (Lawley et al., 2017; Wang et al., 2020) which is in line with the changes observed here during HDT. Notably, changes observed immediately upon entering microgravity (+0.44 cm^2^; Lawley et al., 2017) and in heart failure patients (Pellicori, Kallvikbacka‐Bennett, et al., 2015) are substantially larger and JVA monitoring may show greater reliability in these settings. Future work should emphasize quantifying the impact of each individual factor on the JVA signal as well as the determining the minimal change in blood volume that can be detected by CHI.

The main limitation of the current interpretation of JVA is that factors which may impact the signal due to their influence on absorption characteristics of whole blood, including hemoglobin concentration and venous blood oxygenation saturation (Jacques, 2013), were not measured. The main methodological limitation of this study was that JVA and CSA were not measured on the same side of the neck which could impact the strength the associations. Previous research found the right IJV to be larger than the left, regardless of sex (Bos et al., 2016; Jeon et al., 2020; Magnano et al., 2016); however, simultaneous measurement on the same vessel was not practical given that the presence of ultrasound gel would interfere with the optical reflectance used to quantify JVA. In addition, the interpretation of any individual within‐person correlation coefficient presented here needs to be viewed with a degree of caution as they are based on only 3 or 4 data points. The relatively narrow IQR and moderate to strong median effect sizes for most within‐group correlation coefficients—especially with larger changes in CSA—however, provides some confidence that the association is real. Moreover, the individual correlation coefficients were in general agreement with the repeated‐measures approach which provides an overall reflection of the association bewteen JVA and CSA.

## 5 CONCLUSION

In this study, we identified unique physiological differences between sexes, with men having a greater change in JVA with both cephalid and caudaul shifts, while women exhibit a larger decrease in CSA during LBNP between rest and −40 mmHg. We also observed moderate to strong correlations between changes in IJV CSA and attenuation of near‐infrared light from the IJV supporting a role for CHI as a tool to monitor changes in IJV blood volume during acute changes in central blood volume. This shows preliminary evidence that under controlled conditions and with sex‐dependent interpretation of data, CHI may be a useful technology for non‐contact monitoring of venous congestion in conditions such as heart failure or central hypovolemia during hemorrhagic shock.

## CONFLICT OF INTEREST

R.A. reports patents that are related to the technology described in this study. Data were acquired and processed by co‐authors unaffiliated with any commercial entity.

## AUTHOR CONTRIBUTIONS

C.A.P., R.A., R.L.H., and A.D.R. conceived and designed research; C.A.P., R.A., E.S., H.H., and A.D.R. performed experiments; C.A.P., R.A., E.S., H.H., and A.D.R. analyzed data; C.A.P., R.A., R.L.H., and A.D.R. interpreted results of experiments; C.A.P. and A.D.R. prepared figures; C.A.P. and A.D.R. drafted manuscript; C.A.P., R.A., E.S., H.H., R.L.H., and A.D.R. edited and revised manuscript; C.A.P., R.A., E.S., H.H., R.L.H., and A.D.R. approved final version of manuscript.
